# Extended Genotyping to Stratify the Risk of CIN2+ in Women with Persistent HPV Infection, Negative Cytology and Type 3 Transformation Zone

**DOI:** 10.3390/cancers16101816

**Published:** 2024-05-10

**Authors:** Maria Teresa Bruno, Gaetano Valenti, Antonino Giovanni Cavallaro, Ilenia Palermo, Tiziana Aiello, Jessica Farina, Marco Marzio Panella, Liliana Mereu

**Affiliations:** 1 Gynecology and Obstetrics Unit, Department of General Surgery and Medical-Surgical Specialty, Rodolico University Hospital, University of Catania, 95123 Catania, Italy; ninocavallaro@tin.it (A.G.C.); mpanella@unict.it (M.M.P.); liliana.mereu@unict.it (L.M.); 2 Multidisciplinary Research Center in Papillomavirus Pathology, Chirmed, University of Catania, 95123 Catania, Italy; 3 Humanitas, Gynaecologic Oncology Unit, 95100 Catania, Italy; gaetano.valenti@humanitascatania.it; 4 Virology Unit, Rodolico Polyclinic, 95123 Catania, Italy; palermoilenia77@gmail.com; 5 Section of Anatomic Pathology, Department of Medical Surgical Sciences and Advanced Technologies “G.F. Ingrassia”, University of Catania, Via Santa Sofia 78, 95123 Catania, Italy

**Keywords:** HPV persistence, cytology, type 3 transformation zone, extended genotyping, HPV non-16/18 genotype, p16/Ki67

## Abstract

**Simple Summary:**

Persistently hrHPV-positive women with negative cytology and zone transformation type 3 are at increased risk of being diagnosed with CIN2+. The presence of a type 3 transformation zone, typical of postmenopausal women, makes colposcopy and cytology unnecessary and the use of diagnostic LEEP excessive. Since the prevalence of CIN2+ also appears to affect non-16/18 HPV genotypes, extended HPV genotyping and subsequent use of biomarkers for risk stratification could be a feasible strategy.

**Abstract:**

Persistent human papillomavirus (HPV) infection is recognized as a major risk factor for cervical cancer. Women with persistent HPV and negative cytology are at greater risk of CIN2+ than women with negative infection. The diagnosis becomes more complicated when the woman has a type 3 transformation zone at colposcopy. The aim of this study was to determine the prevalence of CIN2+ in women with persistent HPV, negative cytology and TZ3; how to stratify the risk of CIN2+; and what the best diagnostic strategy is, given TZ3. Methods: In a multicenter retrospective cohort study, we enrolled women with negative cytology and TZ3 among the 213 women referred for colposcopy for persistent HPV. The average age of the women was 53 years; in particular, 83% were postmenopausal women. In the presence of a TZ3, the entire transformation zone cannot be explored, making colposcopy and targeted biopsy useless and inadequate, with great risks of underdiagnosis or missed diagnosis. Women with TZ3 underwent diagnostic LEEP to ensure correct diagnoses. Results: The study highlighted 19% (16/84) of CIN2+ lesions, a higher frequency of non-HPV 16/18 genotypes (76.2%), and 50% of CIN2+ lesions being due to non-HPV 16/18 genotypes. Furthermore, more than half of the women (80.9%) had normal histopathological results in the LEEP sample. Conclusion. Women with viral persistence, negative cytology, and TZ3 have a 19% risk of CIN2+; genotyping helps stratify risk, but extensive genotyping is necessary instead of partial genotyping (16/18), referring to a population of women over 50 years old in which the prevalence of genotypes 16,18 decreases and the prevalence of other genotypes increases; diagnostic LEEP is excessive (only 16 cases of CIN2+ out of 48 cases treated), even though 83% of women had viral clearance after LEEP; p16/Ki67 double staining could be a potential risk marker, which would only highlight women at risk of CIN2+ to undergo LEEP. To individualize the diagnostic workup and treatment and minimize the risk of under diagnosis and overtreatment, future studies should explore the use of extended genotyping and new biomarkers for individual risk stratification.

## 1. Introduction

Cervical cancer (CC) is the third most common cancer in women worldwide, and is the leading cause of cancer death among women in developing countries [1]. Human papillomavirus (HPV) infection is a sexually transmitted disease due to the persistence of high-risk HPV genotypes whose prevalence shows significant differences worldwide [2,3]. In the most recent evaluation by the International Agency for Research on Cancer (IARC), 12 genotypes (HPV16, 18, 31, 33, 35, 39, 45, 51, 52, 56, 58, and 59) were classified as carcinogenic, and HPV 68 and 66 have been classified as probably carcinogenic to humans (group 2A). Therefore, several HPV types have been characterized as oncogenic, but not all have the same carcinogenic potential; HPV16, in particular, has shown unique carcinogenic potential. The genotypes that confer greater persistence with progression are 16, 31, and 33, which are very frequent in Italy, while HPV 16, 33, 31, and 18 are more frequent in the United States [4,5,6]. A 2008 meta-analysis confirmed that the persistence of high-risk HPV is significantly associated with the development of high-grade lesions which, if not treated in time, can progress to cervical cancer [7]. Genotypes 16 and 18 are the most oncogenic, with HPV 16 alone being responsible for more than 50% of cervical cancers, and both causing 70% of all cervical cancers [8]. Due to the treatment of precancerous lesions, the implementation of screening with HPV testing, colposcopy, and HPV vaccine, it is hoped that cervical cancer can be defeated by 2030 [9,10,11,12,13,14]. The World Health Organization has set a global goal of eliminating cervical cancer, defined as achieving an incidence of fewer than 4 cases per 100,000 women per year [15,16].

HPV infection is highest in younger women (<25 years old); therefore, a decreasing trend is observed with increasing age. Adolescents and young women easily achieve viral clearance after 18 to 24 months. Persistence of the virus, on the other hand, is typical in women over 25 years of age. Viruses with a high oncogenic risk (hrHPV) are those capable of persistence. The persistence of high-risk genotypes identifies women at risk of preneoplastic lesions that, if not diagnosed and treated in time, can progress to cervical cancer [17,18].

Persistent infection has been defined as the detection of the same type of HPV at ≥2 consecutive visits spaced ≥6 or ≥12 months apart. In this case, the use of genotyping is important as it can highlight the persistence of the specific genotype, HPV 16, which represents the genotype with the greatest risk of persistence. American guidelines recommend that HPV-16-positive women undergo colposcopy even in the presence of normal cytology [19].

HPV testing has a higher sensitivity in detecting CIN2+ than cytology, and is currently recommended as the primary screening method for cervical cancer in Italy [20]. Cytological triage is recommended for HPV-positive women. If the HPV repeat test after one year is still positive, the patient would persistently HPV-positive and exhibiting negative cytology; the optimal clinical management of such women is still not clear. The literature highlights how women with normal cytology and persistent hrHV have a much higher risk of developing CIN2+ than HPV-negative women. Long-term follow-up of the Swedescreen study showed that 100% of cytology negative women with a persistence of genotypes specific HPV developed CIN2+ within 7 years [21]. Other authors have found a 5-year risk of CIN3+ of 7.4% in women with persistent HPV infection and normal cytology [22]. The clinical situation is complicated in women over 50 years of age, at which time both cytological triage and colposcopy have a lower sensitivity for the detection of CIN2+ [23]. The cervixes of postmenopausal women undergo a series of secondary changes due to the lack of estrogen; the transformation zone extends into the cervical canal, making it difficult to explore and constituting the type 3 transformation zone (TZ3), according to the nomenclature of the International Federation of Cervical Pathology and Colposcopy of 2011 [24].

The management of women with persistent HPV infection, negative cytology, and type 3 transformation zone is an unresolved clinical problem. The guidelines are to leave their management to the experience of the colposcopists, and several alternatives are possible: local treatment with estrogen, repeat endocervical tests, biopsies with random punch, or diagnostic conization. However, the most popular route is diagnostic LEEP, which ensures a correct diagnosis [25,26]. We therefore studied the outcomes of women undergoing LEEP for type 3 transformation zone, persistent HPV, and normal cytology in order to determine the prevalence of CIN2+ and the usefulness of extended genotyping for risk stratification.

## 2. Materials and Methods

From the dedicated database, the anonymized data of 213 women were studied. These women had been sent for colposcopy due to HPV persistence to the second-tier center of the University Hospital of Catania and the Humanitas center of Catania from 2019 to 2022. Of these, we selected women with negative cytology and who had undergone diagnostic LEEP for type 3 transformation zones. We studied the clinical cases of which we were able to reconstruct the entire diagnostic and therapeutic process from colposcopy to LEEP and which met the following inclusion criteria: unvaccinated women who had undergone hrHPV genotyping before and after LEEP, diagnosis of a type 3 transformation zone on colposcopy, negative cytology at baseline, and the state of the cone margins was clear as well as the possible presence of any residual disease. Exclusion criteria were as follows: women undergoing LEEP for CIN, women who had no follow-up data, who had a history of cervical cancer or HIV or other conditions affecting their immune response. Residual disease is defined as a diagnosis of CIN2+ at the first post LEEP valuation, and recurrence is defined when the CIN2+ lesion is diagnosed after a negative test. Persistent infection has been defined as the detection of the same genotype of HPV at ≥2 consecutive visits spaced ≥6 or ≥12 months apart. Incident or transient HPV infections are defined as the detection of a new HPV genotype.

The follow-up protocol involved repeating the co-test followed by colposcopy every six months for two years and then annually; if three subsequent co-tests were negative, it was repeated every three years.

A ThinPrep PreservCyt cervical specimen (Hologic, Inc., Bedford, MA, USA) was collected from all patients at baseline and post LEEP for HPV DNA testing and HPV genotyping.

The cervical cytological examination was interpreted using the Bethesda system, and the World Health Organization (WHO) classification was used for the histological diagnosis of the removed specimens.

Both included centers used the same molecular technique for the detection and genotyping of HPV DNA. HPV types were classified as high-risk using the guidelines from the International Agency for Research on Cancer. The identified genotypes were divided into HPV 16, HPV 18, and non-HPV 16/18 genotypes (HPV 31, 33, 35, 45, 39, 51, 52, 58, 59, 66, and 68). Only 95 patients met the inclusion criteria and their clinical data were collected: age, HPV genotype, and histological cone examination. We studied the margins of the histological cone resection and the presence of any residual disease.

The data were analyzed anonymously. This study complies with the provisions of the Declaration of Helsinki, as revised in 2013. The study protocol was notified, in accordance with the current legislation on observational studies provided by AIFA, to the Ethics Committee of the University Hospital of Catania, which did not request additions or changes to the protocol. In addition, the Ethics Committee of the University Hospital of Catania considered the consent of the study participants to be unnecessary as the study concerned only a retrospective review of the medical database.

### 2.1. HPV DNA Testing and HPV Genotyping

Exocervical cytologic samples were taken and placed in ThinPrep solution. Samples were sent to the laboratory for DNA extraction and viral DNA genotyping by genetic amplification followed by hybridization with genotype-specific probes capable of identifying most HPV genotypes of the genital region; high-risk HPV genotypes (16, 18, 26, 31, 33, 35, 39, 45, 51, 52, 53, 56, 58, 59, 66, 68, 73, and 82), low risk (6, 11, 40, 43, 44, 54, and 70) and indefinite risk (69, 71, and 74). The commercial method used was the MAG NucliSenseasy system (bioMerieux SA, Marct l’Etoile, France). HPV testing was performed using a previously reported method [27]. We divided genotypes into HPV 16, HPV 18, and non-HPV 16/18 hrHPV.

### 2.2. Colposcopy

Colposcopy was performed using a Zeiss OPM1F colposcope (Carl Zeiss, Jena, Germany) and applying acetic acid and a Lugol iodine solution. Any colposcopic abnormality was classified according to the nomenclature proposed by the International Federation for Colposcopy and Cervical Pathologies (IFCPC) into 3 grades of increasing abnormalities depending on severity: abnormal transformation zone (ATZ) grade 1 (ATZ1), grade 2 (ATZ2), or cancer. We evaluated the visibility of the squamous–columnar junction and the transformation zone, which can be type 1, 2, or 3 if the squamous-columnar junction is fully visible, fully visible but enters the canal, or not visible when it is in the canal and where it ends cannot be seen, respectively; this classification correlates with the type of excision.

### 2.3. LEEP Technique

LEEP was performed with colposcopic guidance under local anesthesia in the outpatient clinic by experienced staff; with type 3 transformation zones, we used a Fisher loop whose size was adapted to the volume of the cervix. We obtained a single cone with a length of between 15 and 20 mm, to be sure of including the entire transformation zone in the excision. Histology of the cervical cone was established, and involvement of the cone margins was assessed. Cone margins were positive if the distance between CIN2+ and the resection surface was <1 mm.

### 2.4. Statistical Analysis

Statistical analysis was performed using the SPSS software package for Windows (version 15.0, SPSS, Chicago, IL, USA). Descriptive statistics are expressed as the frequency, arithmetic mean, and percentages. The results are summarized in the tables. The relationship between categorical variables was assessed using Chi-squared tests or Fisher exact tests, depending on the sample size. Contingency tables were created to compare the prevalence of CIN2+ between HPV-16/18-positive women and women positive for non-16/18 HPV genotypes. Values with *p* < 0.05 were considered statistically significant.

## 3. Results

**Figure 1:**
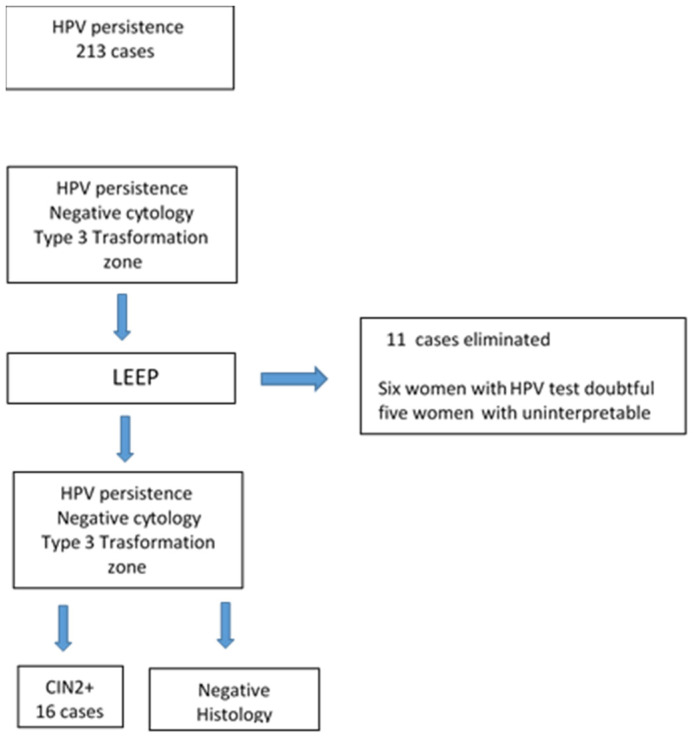
Flow-chart of the study population.

Of the 95 women enrolled, 11 women were excluded: 5 women had doubtful HPV testing and 6 women had uninterpretable cone margins. The final study sample consisted of 84 women (Figure 1).

The mean age of the women was 53 years (range 41–77); 70/84 (83%) women were postmenopausal.

Thirteen different genotypes were identified; genotypes 16/18 were responsible for 24 (28.5%) HPV-infected cases, while non-HPV genotypes 16/18 (HPV 31/33/45/35/39/51/52/58/59/66/68) were responsible for 60 (76.2%) cases of infection. The most represented genotype was HPV 16, with 18 cases; followed by HPV 31, with 16 cases; then HPV 33, with 8 cases; genotypes 18/35/39/52/59, with 6 cases; HPV 45 and HPV 52, with 4 cases; HPV 66, with 2 cases; and HPV 58 and 68, both with 1 case.

Analyzing the histological examination of the cone, 50 (59.5%) cases were negative, 18 (21.4%) cases had CIN1, while 16 (19%) cases were diagnosed with CIN2+ (Table 1).

The 16/18 genotype was responsible for 50% of CIN2+ cases (8/16); the other eight cases of CIN2+ were due to non-16/18 genotypes: 31/35/52/59. Persistent infection with non 16/18 HPV genotypes is significantly associated with CIN2+ (*p* = 0.03) (Table 2).

None of the LEEP samples showed invasive disease, although 6 of the 16 CIN2+ excisions had positive endocervical margins. Analysis of viral status after LEEP revealed that 64/84 (76.2%) women had resolved their HPV infection, while 20/84 (23.8%) women still had a persistent HPV infection.

The HPV types persisting after LEEP were HPV 16 (*n* = 8), genotype 31 (4 cases), and genotypes 18/51/52 (2 cases). The study of the cone margins revealed only six cases of positive endocervical margin, in one of which there was residual disease and HPV persistence. In the latter case, the woman was subjected to a second LEEP. The other four cases were negative for the virus, with no residual disease, and underwent follow-up. After three years of follow-up, all were negative and disease-free. Two cases with a positive esocervical margin but no residual disease underwent follow-up.

## 4. Discussion

The present study with a result of 19% CIN2+ in the study sample highlighted how women with persistent HPV and negative cytology are at risk of CIN2+ lesions. This finding is in line with other studies that found a 15% to 25% risk of CIN2+ in women with persistent and negative cytology [26,28]. Recently, this concept was reaffirmed by the Swedescreen study, in which 100% of women with persistence of HPV-type-specific and negative cytology developed CIN2+ within 7 years. In particular, the long-term follow-up of the Swedescreen study strongly supports that persistent infection, especially type 16, is one of the most important clinically relevant risk factors for the occurrence of CIN2, even in women with negative cytology [21].

Clinical management of HPV positive and cytology negative women involves repeat HPV testing 12 months later and, if a persistence of type-specific HPV is found, referral for colposcopy with targeted biopsies and endocervical cytology [29]. Management problems arise in cases where colposcopy shows a type 3 transformation zone, which is typical post menopause, and the entire transformation zone is not explorable, making targeted biopsy useless and inadequate with great risks of underdiagnosis or non-diagnosis [26,30]. In women with type 3 transformation zones, it is difficult to obtain adequate amounts of tissue from the endocervix, even with endocervical curettage (ECC), whereas cytologic sampling with a cytobrush has sensitivity in detecting lesions that ranges between 44% and 93% [31]. The clinical management of women with persistent HPV, negative cytology, and type 3 transformation zones is difficult.

Arnio et al. investigated the clinical management of these women by comparing the results of LEEP and targeted biopsy. They reported CIN2+ in 15% of women with LEEP, while biopsies failed to detect any cases of CIN2+ [23]. As a result, several guidelines suggest the use of LEEP in women with viral persistence and type 3 transformation zones on colposcopy, to ensure a correct diagnosis.

The present study subjected the 84 “women” to LEEP, obtaining 19% CIN2+ and a post-LEEP clearance in 76% of the cases studied. These results make LEEP appear excessive in the treatment of the study sample (only 16 cases of CIN2+ out of 84 cases treated), even if it appears to be an excellent means of preventing CIN2+, given the high percentage of viral clearance (76%; 64/84 cases) obtained following the primary LEEP [32,33].

The results of the present study show that 19% of women with negative cytology and HPV persistence have CIN2+, including patients with non-16/18 HPV strains. This is consistent with existing data showing that cytology has poor sensitivity in postmenopausal women, while HPV is independently indicative of high-grade dysplasia [20,34].

Examination of the genotypes of the sample under study showed a variability of genotypes. In the present study, 83% were postmenopausal women (type 3 transformation zone); there was a higher frequency of non-HPV 16/18 genotypes (76.2%) and 50% of CIN2+ lesions were due to non-HPV 16/18 genotypes. The discovery of CIN2+ lesions associated with non-HPV 16/18 genotypes (HPV 31/39/51/52/59) confirms that in women over 50 years of age, the frequency of specific genotypes is different from that of women of childbearing age [35,36,37].

These findings are consistent with previous studies in which women with HPV-16- or HPV-18-positive cervical cancer tended to be younger at diagnosis than women positive for non-16/18 genotypes [38,39]. The observed age-specific difference in genotype-specific prevalence could be the result of a more rapid progression to HPV-16- and HPV-18-positive precancerous lesions compared to precancerous lesions attributed to other cancerous HPV genotypes. This may be because HPV 16 and 18 are more commonly integrated into the host genome, while non-16/18 genotypes are more likely to be episomic [40,41].

In the present study, persistent infection with non-16/18 HPV genotypes was significantly associated with CIN2+ (*p* = 0.03); these data support the use of extended genotyping for this target group of women.

In postmenopausal women, the oncogenic potential of each genotype is less studied because non-HPV 16/18 hrHPV genotypes are often tested as a pool (partial genotyping); using extended HPV genotyping would highlight the specific non-HPV 16/18 genotypes by exploring the CIN2+ risk of each individual genotype.

Data from the present study show that more than half of the women (80.9%) had a normal histopathological result in the LEEP sample; therefore, offering LEEP to all women in the target population increases the risk of overtreatment. The management of these cases could be supported by the use of biomarkers. Dual p16/Ki67 staining could be a potential risk marker, as studies have shown that it has a higher sensitivity and negative predictive value for the detection of CIN2+ compared to cytology in screened younger female populations [42,43]. Not much is known about the clinical use of dual p16/Ki67 staining in populations of women over 50 years of age. A recent study showed that double-stained cytology shows high sensitivity and specificity profiles for CIN2+ in postmenopausal women [44,45]. Previous studies have suggested a protective role of the HPV vaccine in women who already had an HPV infection, although the exact mechanism of this phenomenon and its clinical impact have not been fully clarified [46,47].

Limitations of this study are the small sample size, which reduces the power of interpretation, and its retrospective design. Another limitation could be represented by the origin of the study population from a second level center; thus, the results may not be adequate for the general population. The strengths of the study are the homogeneous sample, the multicentric nature, and the centers that used the same technology for genotyping; the use of extensive genotyping that allowed us to identify the genotypes responsible for CIN2+ in postmenopausal women.

## 5. Conclusions

Persistently hrHPV-positive women with negative cytology and TZ3 are at increased risk of being diagnosed with CIN2+. In a population of women over 50 years of age, the prevalence of genotypes 16,18 decreases and the prevalence of HPV non 16/18 genotypes increases. Since the prevalence of CIN2+ also appears to affect non-16/18 HPV genotypes, extended HPV genotyping and the subsequent use of biomarkers for risk stratification could be a feasible strategy.

## Figures and Tables

**Table 1:** Genotype, viral status, age, margin status, and residual disease in the 16 CIN2+ cases.

Genotype	Viral Status	Age	Endocervical Margin	Esocervical Margin	Residual Disease	FU
HPV 16	Persistence *n* = 4	43	Negative	Positive	no	FU
		42	Negative	Positive	no	FU
		51	Positive	Negative	si	second LEEP
		49	Negative	Negative	no	FU
	Clearance *n* = 2	41	Positive	Negative	no	FU
		57	Negative	Negative	si	FU
HPV18	Persistence *n* = 2	41	Positive	Negative	no	FU
		42	Negative	Positive	no	FU
HPV31	Clearance *n* = 2	61	Negative	Negative	no	FU
		56	Negative	Negative	no	FU
HPV 33	Clearance *n* = 1	46	Negative	Negative	no	FU
HPV 35	Clearance *n* = 2	56	Negative	Negative	no	FU
		62	Negative	Negative	no	FU
HPV 52	Persistence *n* = 2	65	Negative	Negative	no	FU
		58	Negative	Negative	no	FU
HPV 59	Clearance *n* = 1	49	Negative	Negative	no	FU

**Table 2:** Odds ratio for CIN2+ for 16/18 HPV genotypes and non-16/18 HPV genotypes.

Genotypes	CIN2+	OR CI 95%	*p* Value
HPV16/18	8/24	3.25 (1.05–10.05)	0.03
HPV non 16/18	8/60	0.3 (0.10–0.95)	0.03

## Data Availability

Data are contained within the article.
